# A hybrid BiLSTM and rule-based system for integrated diabetes prediction and personalized guidance

**DOI:** 10.1371/journal.pone.0347672

**Published:** 2026-05-22

**Authors:** Muhammad Saleem, Muhammad Hamid, Saadia Malik

**Affiliations:** 1 Department of Industrial Engineering, Faculty of Engineering, King Abdulaziz University, Rabigh, Saudi Arabia; 2 Department of Computer Science, Government College Women University, Sialkot, Pakistan; 3 Department of Information Systems, Faculty of Computing and Information Technology, King Abdulaziz University, Rabigh, Saudi Arabia; Institute of Management Sciences Peshawar, PAKISTAN

## Abstract

Diabetes is a common chronic disease that needs early diagnosis and proper management to avoid severe complications. While current Artificial Intelligence (AI) tools generate predictive information, they often lack an integrated element for post-diagnosis support in order to fill in this critical gap in patient self-management. This research proposes and validates a hybrid System which aims to bridge this gap. The methodology is based on a novel, fused dataset (PIMA and Type 2 Diabetes) that was carefully preprocessed following a leakage-safe protocol in order to increase generalizability. The system architecture is a combination of two different critical components: Strong Bidirectional Long Short term Memory (BiLSTM) model for prediction and rule based engine for creating personalized lifestyle recommendations. In order to validate the efficacy of the BiLSTM model, seven traditional machine learning (ML) models and standard deep learning (DL) models have been comparatively tested, in which BiLSTM model has demonstrated a better generalization and prediction performance. Rigorous 10-fold cross validation was used to validate the system, which came up with an accuracy of 84.02%, precision of 87.89%, and recall of 80.50%. This research concludes that by successfully combining the high-performance predictive engine and real-time guidance module, it is possible to develop a holistic clinically relevant tool to close the loop between diagnosis and proactive self-management.

## 1. Introduction

Diabetes is a globally widespread and critical disease affecting millions of people and associated with increased blood sugar levels (hyperglycemia). The two main types of diabetes, Type 1 and Type 2, have different causes and mechanisms: Type 1 diabetes is an autoimmune condition where the pancreas is unable to produce insufficient or no insulin, by far the more common form of diabetes, is caused by an inability of the body to effectively use the insulin produced by the pancreas. The statistic reveals that there will be more than half a billion people living with diabetes in different parts of the world by 2030 [1]. Moreover, it is also estimated that 240 million individuals have undiagnosed diabetes and almost half of all adults lack knowledge of the condition. The long-term effect of improper control of diabetes is devastating and can result in cardiovascular diseases, kidney malfunction and neuropathy [2].

Some of the common symptoms to identify diabetes are frequent urination, elevated thirst and hunger, unwanted weight loss, fatigue and blurred vision. This latency represents an important need for early and accurate detection, but traditional invasive glucose monitoring has poor patient compliance because of associated discomfort [3]. Consequently, there is a strong need for accessible and non-invasive diagnostic tools that will guarantee consistency of health monitoring.

The advent of ML provided a powerful alternative to this and the focus has moved from reactive diagnosis to proactive risk assessment using algorithms such as Support Vector Machine (SVM) and Random Forest [4]. While good on static datasets, these models have a basic flaw in that they analyze health data as discrete events and not continual timelines. Chronic diseases develop gradually and the conventional methods of ML typically overlook important patterns over time like reading a single frame of a video and not knowing the plot. This failure to interpret longitudinal health trends requires more sophisticated architectures that are able to capture the dynamic trajectory of the health of a patient [5].

In order to fill this gap, this paper proposes a novel, integrated system that utilizes a Bidirectional Long Short-Term Memory (BiLSTM) model. Unlike the standard classifiers, BiLSTM processes information bidirectionally, which allows it to capture complex, non-linear inter-feature relationships which are crucial for the accurate early prediction. However, prediction is not sufficient. Therefore, the proposed system integrates this advanced predictive model with a rule-based DSS. This hybrid methodology is able to use the predictive information gained from the BiLSTM model to generate actionable and individual lifestyle recommendations bridging the gap between risk identification and patient self-management. The key contributions of this study are as follows:

1. The design and implementation of BiLSTM model, which was designed to capture the complex non-linear dependencies of clinical data for robust diabetes risk assessment.
2. Integrated BiLSTM predictive engine with a rule-based recommendation module as a working web-based prototype demonstrating a clear path from diagnosis to personalised patients self-management.

To guide the development and validation of this system, this research answers the following significant questions:

1. ***To what extent can a hybrid DL architecture as a combination of BiLSTM model and rule-based engine improve the accuracy and clinical utility of diabetes risk prediction models compared to multi-model models?***
2. ***How can a high accuracy predictive diagnosis be translated effectively into actionable and personalised guidance to bridge the gap between risk identification and patient self- management?***

The remaining paper proceeds as follows: Section 2 provides a review of related literature, Section 3 outlines the proposed research methodology, Section 4 explains the results and discussion, Section 5 concludes the study and suggests directions for future work.

## 2. Literature review

The use of AI in healthcare has sparked a significant body of research on how to predict diabetes, where we see a certain linear, evolutionary path going from simple applications of ML to complex integrated health management systems. Initial research focused mostly on determining the predictive capabilities of different traditional ML algorithms. A representative study by Ojha and Bajpai [6] analyzed a panel of classifiers, such as SVM, Random Forest, using electronic health record (EHR) data, which led them to conclude from their comparative study that the most effective classifier in diagnosis was the Gradient Boosting classifier. While fundamental, the main drawback of this research was to determine the most suitable and superior single model and consequently the superior potential of ensemble techniques relatively unexplored.

This gap was filled out by a new wave of research that proved how powerful the combination of models can be. For example, Hasan et al. [7] have proposed an advanced framework based on a broad preprocessing pipeline and a weighted ensemble of AdaBoost and XGBoost classifiers on PIMA Indians Diabetes dataset, from which the state-of-the-art accuracy of 0.950 was derived in terms of AUC. The height of this method was revealed by Saihood and Sonuç [8] that showed a three stage method of extensive preprocessing and stacked ensemble of Random Forest and SVM using PIMA dataset that got state of art accuracy of 97.50%. This high level of performance was echoed by Ganie et al. [9] whose comparative study of boosting algorithms on the PIMA dataset showed that a fine tuned Gradient Boosting model could achieve a high level of accuracy of 96.75%. The difficulty in using these methods on more diverse data was illustrated by Dutta et al. [10] whose weighted ensemble method on a novel data set from South Asia population showed a more modest 73.5% accuracy, and thus highlights the importance of robust preprocessing. However, despite their impressive accuracy, all these studies share a common and critical limitation- their function is limited to that of diagnosis, with a highly accurate risk score but without any integrated component to guide the patient as to what to do next, basically resulting in a ‘diagnostic dead end.’

To beat the limitations of traditional ML such as the requirement for manual feature engineering, researchers started taking a look at DL. Rabbani et al. [11] presented an early comparison, using both the standard Artificial Neural Network (ANN) and the Convolutional Neural Network (CNN) based hybrid model for a large-scale UCI Diabetes dataset, showing that the CNN model gave a better accuracy of 92.4%. To better deal with the temporal nature of health data, Shams et al. [12] proposed a hybrid RFE-GRU model using Recursive Feature Elimination (RFE) in order to obtain key features of the PIMA dataset, and Gated Recurrent Unit (GRU) for training and achieved 90.70% accuracy. The apex of DL’s predictive power was demonstrated by Xiong et al. [13] who proposed a multi-modal DL strategy with an attention-based fusion mechanism to predict Type 2 Diabetes complications. Evaluated with incredibly large dataset of 15,000 patients, their system was able to prediction with a 94.7% accuracy. Yet, even these highly sophisticated DL models largely inherited the same limitation of their ML predecessors their primary contribution was limited to prediction, without bridging the crucial gap to manage patients. Almutairi et al. [14] adopted a strategy, where they employed statistical and ML algorithms such as ANFIS to predict the occurence of diabetes in Saudi Arabia, which is a crucial task to inform the policy of the population, not to diagnose a particular patient. Meanwhile, Khokhar et al. [15] focused on the critical aspect of transparency, applying Explainable AI (XAI) with a SMOTE ensemble model to the Diabetes Binary Health Indicators dataset, achieving a high accuracy of 92.5% with the identification of key predictors. This increases clinical trust though the system remains a standalone predictive tool.

Recognizing that a prediction is only useful if it is acted on a second stream of research emerged: looking to build integrated, patient-centric systems. Sajid et al. [16] have proposed one of the first truly hybrid paradigms, in which the authors have used an optimized ML ensemble (combination of RF, SVM, and XGBoost) for accurate prediction, which on diagnosis, activated a comprehensive food and exercise, however, its primary weakness lies in its predictive engine; by relying on traditional ML ensemble models, it lacks the capacity for deep, automatic feature extraction offered by more advanced DL architectures. This trend of focusing on the management component has also been observed in the work of Sowah et al. [17] building an end-to-end system consisting of Tensorflow-based neural network for food recognition and KNN model to recommend meals. The clinical utility of such AI-driven management has been empirically proven by Reddy [18] whose quasi-experimental study revealed an AI-assisted therapy group had significantly improved health outcomes compared to those receiving the standard care. Furthermore, Sivaramakrishnan et al. [19] developed a system for HbA1c management empowered by AI that leveraged wearables and a collection of ML algorithms, such as LSTM, to provide individualized guidance. The limitation in these management-focused systems is often the inverse of the first stream: that the recommendation logic takes precedence and often results in the lack of use of a really state-of-the-art predictive model for the initial diagnosis.

A systematic review of the existing literature shows a clear and undying gap in the field of AI- based diabetes prediction. The research has largely broken into two different streams, the one leading to highly accurate and state-of-the-art predictive models, which used as stand-alone diagnostic tools, and the other to integrated management systems, while offering useful information for guiding patient management they often lack the most sophisticated predictive engines. Therefore, one central research gap remains lack of a single framework that will guarantee a high degree of predictive fidelity and providing actionable patient-centric advice at the same time. This gap is directly covered by this research as it offers a hybrid system combining an effective BiLSTM model, which provides high accuracy prediction, and a rule-based system, which provides personalized recommendations.

## 3. Research methodology

**Fig 1:**
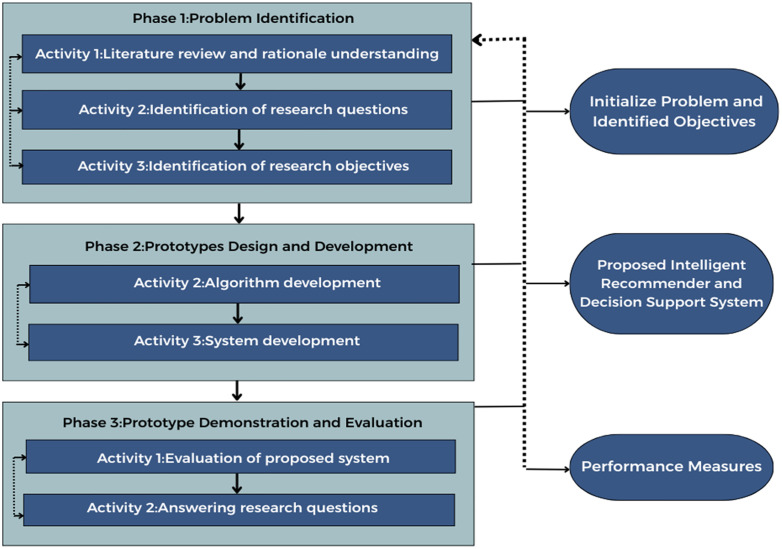
The design science research methodology flowchart.

This study utilized the Design Science Research (DSR) methodology, which is a structured methodology for the creation and evaluation of Information Technology (IT) artifacts used to solve real world problems. The DSR methodology for this study consist three different phases: (1) problem identification, (2) prototype design and development, and (3) prototype demonstration and evaluation. This systematic methodology is the only way to ensure that the resulting system is not only technically sound but clinically relevant and effective as well. The flow diagram of the proposed methodology is presented in Fig 1.

### 3.1 Problem identification phase

The first phase in this research has revealed basic issues that centered on accurate prediction of diabetes risk, effective patient guidance. A review of the existing literature, presented in Section 2, indicated two main and interrelated problems to be solved by this study. First one is that traditional ML models can often have limited ability to capture more complex, non-linear dependencies in diverse clinical data, which can limit their ability to provide an accurate diagnosis. Second, existing research is often limited to diagnosis with a risk score, but does not translate this into immediate and actionable patient self-management strategies. This dual-problem of needing more sophisticated predictive model and solid intervention framework set a straightforward justification for this research which consists of forming a complete fused data set then developing a state-of-the-art model BiLSTM in order to leverage its unique traits of understanding relations between features for effective diabetes management.

### 3.2 Prototype design and development phase

The second DSR phase was focused on the creation of the integrated DSS artifact. This was achieved through two key activities: Algorithm Development, which involved preparation of the dataset and building the intelligent prediction-recommendation engine, and System Development, where the developed intelligent prediction-recommendation engine was integrated into a practical and easy-to-use web application.

#### 3.2.1 Data collection and preparation.

The foundation of any strong AI model is good and large quantity of data. Recognizing the shortcomings of using only one, independent source, this research used the data-fusing method to build up a larger and generalized amount of data. Two public sources were obtained and integrated: well known PIMA Indians Diabetes Dataset 768 instances and an additional Type 2 Diabetes Dataset 466 instances with a total of 1,234 patient records in our repository. The process of fusion included a strict feature harmonization phase in order to achieve a consistency between the two sources. Harmonization was facilitated by the fact that both data sets used exactly the same clinical parameters that were measured in exact standard medical units (e.g., Glucose in mg/dL, Blood Pressure in mm Hg and BMI in kg/m 2). A direct one to one feature mapping was performed for the following eight feature: Pregnancies, Glucose, Blood Pressure, Skin Thickness, Insulin, BMI, Diabetes Pedigree Function and Age. Due to the inherent consistency in feature space and measurement scales, the datasets were merged without any complicated unit conversions and the original clinical integrity of the data was maintained. Any possible changes in the distribution of data caused by different sources were handled by performing fold wise statistical normalization during the preprocessing stage, which resulted in a cohesive input space for the BiLSTM model.

a) **Data preprocessing**

Following the data fusion, a leakage safe data preprocessing pipeline was set up to guarantee the data is clean, balanced and ready for powerful model training and evaluation. In order to avoid information leakage from the validation set to the training set, all the preprocessing steps such as imputation, resampling and scaling were implemented within each fold of the 10 fold cross validation process. First, to prevent the loss of important clinical information, missing values were imputed fold-wise. For some features such as Glucose and BMI, median imputation was selected due to its robustness to outliers while KNN imputation [20] was used for other features. Notably, the imputation parameters were only estimated from the training subset of each fold and then used for the validation subset. Second, the dataset had a significant class imbalance, which can cause the system to be biased towards the majority (non-diabetic) class. To overcome this, the SMOTEENN (Synthetic Minority Over-sampling Technique with Edited Nearest Neighbors) method was implemented on the training subset of each fold only. This ensured that the model was trained in a balanced and clean feature space without contamination from any validation data which was original and unseen by the model. Finally, since numerical features, such as age, glucose, and BMI, contain different ranges, only the StandardScaler was fit to the resampled training fold in order to normalize the data. This critical step, which ensured a mean of 0 and standard deviation of 1 [21] for all features, was then applied on the validation subset, which “ensured equal contribution of each fold engaged in the learning process” without breaking the principle of statistical independence.

b) **Feature representation**

The methodology for feature representation is entirely based on the implicit capability of the DL model to directly learn from the clinical data’s of the patient. Instead of manual engineering of features, the goal in model design is to automatically find and to weigh the most important predictive patterns. This is achieved by considering the input feature vector as a short sequence and processing it using a BiLSTM. This layer serves as a highly non-linear feature extractor, learning complex and possibly interactive relationships between the clinical variables (e.g., how BMI and Glucose levels interact) that a standard dense model might not learn. This system has the advantage of enabling the model to do its own internal, data-driven representation of features.

c) **Model development**

The key element of this research is the development of a hybrid system that incorporates two main modules:

1. The BiLSTM prediction model

The main part of the system is a BiLSTM model. The architecture is composed as following sequential layers. Its Input Layer accepts the preprocessed patient data in the form of sequence of length 8. These two stacked BiLSTM layers with 128 and 64 units process these sequence in both directions. This is necessary to capture the entire context of the relationships between features. L2 regularization is added so that the network does not overfit. The output from BiLSTM layers is passed to a dense layer having 64 number of units and ReLU activation function. There is the final layer which involves a single neuron with sigmoid activation for predicting the probability of diabetes.

2. The rule-based recommendation engine

This module is activated after the prediction is made. It is a non ML component that employs a set of predefined rules based on established clinical guidelines. It is able to analyze the original input values by the user such as high BMI, high Glucose and the final prediction to deliver personalized, actionable recommendations for diet, exercise and mental wellness.

#### 3.2.2 Algorithm for prediction and recommendation.

The proposed algorithm is below.

**Input:** Patient Health Dataset (diabetes.csv)

**Output:** Diabetes Prediction (0 or 1) & Personalized Recommendations

**Begin**

1.**Load dataset** → diabetes.csv

2.**Data Sanitization →**clinical_features {0}→NaN

3. **Partition dataset** → StratifiedKFold (k = 10)

4. **For each fold i in 1–10:**

   a. **Split raw data →** training_set (9 folds), validation_set (1 fold)

   b. **Leakage-Safe Imputation** → Fit imputer on training_set → Transform validation_set

   c. **Class Balancing →** Apply SMOTEENN exclusively to training_set

   d. **Feature Scaling** → Fit StandardScaler on training_set → Transform both sets

   e. **Data Reshaping →** Convert sets to 3D tensor (shape = samples, 1, 8)

   f. **Define Model Architecture M:**

   **- Input Layer (1, 8)**

   **- BiLSTM (128 units) →** Dropout (0.35)

   **- BiLSTM (64 units, ReLU) →** Batch Normalization

   **- Dense (64 units) →** Dropout (0.35)

   **- Dense (1 unit, Sigmoid) →** y_prediction

   g. **Compile & Train Model M** → Optimizer: Adam → Loss: Binary Cross-Entropy

   h. **Evaluate Model** → Test on validation_set → Compute {Acc, Prec, Rec, F1}

   i. **Store performance metrics**

5. **End For**

6. **Compute overall performance** → mean(Accuracy, Precision, Recall, F1)

7. **Finalize Production Model** → Train M_final on full dataset using leakage-safe pipeline

8. **Define Prediction Pipeline** (new_patient_data)

   a. **Preprocess new_patient_data →** scaled_input

   b. **M_final.predict(scaled_input) →** c_pred ∈ {0,1}

   c. **Generate Recommendations (Rule-Based Engine) →** Recs

   d. Return (c_pred, Recs)

**End**

#### 3.2.3 System development.

In order to illustrate the practical implementation, the system was deployed in the form of a web-based prototype on a client-server architecture. The front end was developed in HTML, CSS and JavaScript to collect user information and show the results, whereas the backend was managed by a Flask server. The core logic is located in the server, and the pre-trained BiLSTM model and StandardScaler are loaded in memory to make immediate predictions and activate the rule-based recommender engine in time.

**Fig 2:**
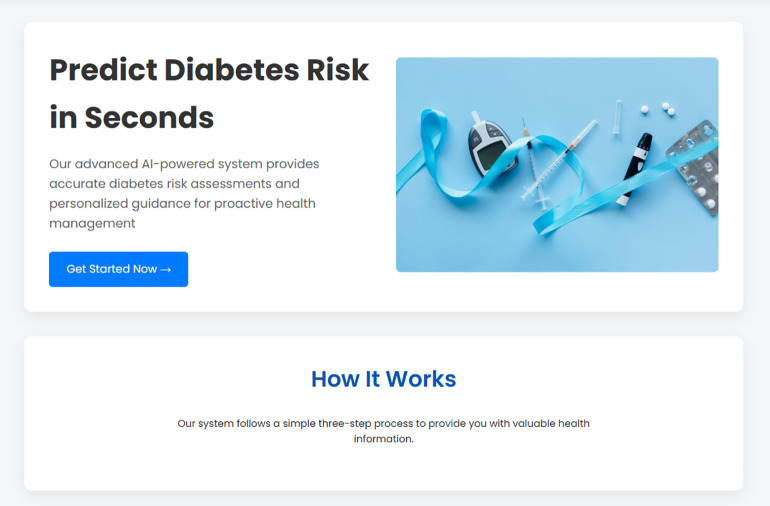
The main user interface of the SugarWise web application.

**Fig 3:**
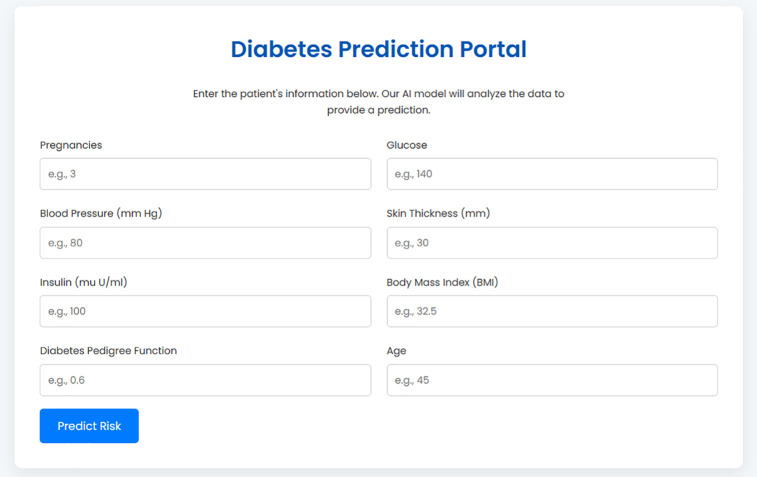
The Data Entry Portal for the BiLSTM Prediction Model.

**Fig 4:**
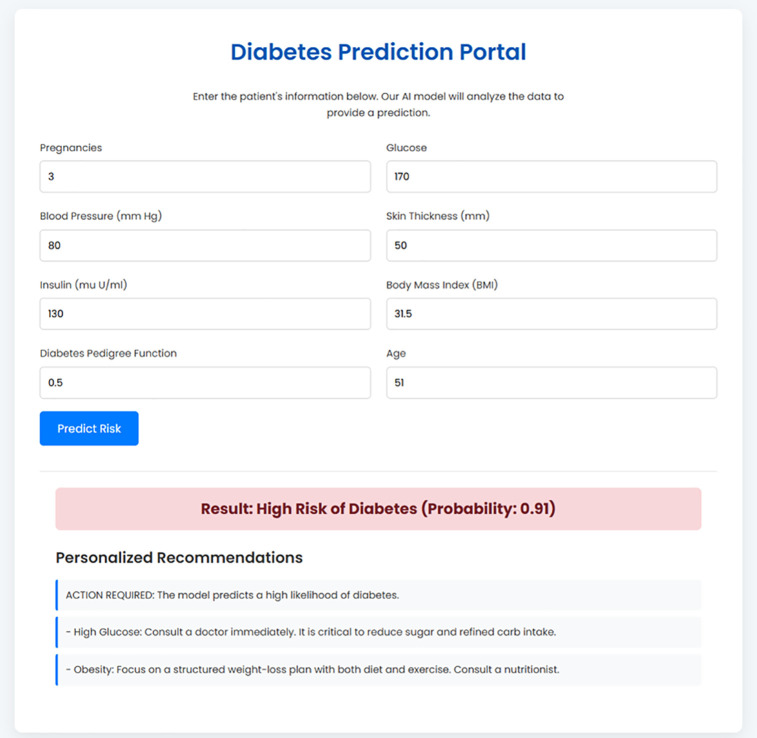
Example of a Positive Prediction Result with Personalized Recommendations.

**Fig 5:**
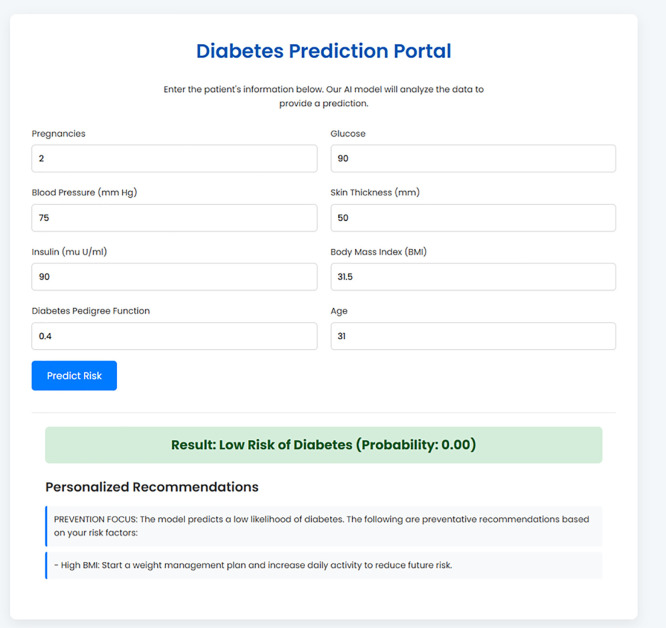
Example of a Negative Prediction Result with Preventative Guidance.

A user enters their key clinical data, such as glucose and BMI, into a clean data entry form as shown in Fig 2 and Fig 3. Upon submission, the backend handles this information and performs one of two results which will then be returned back directly to the interface. A high-risk prediction is associated with personalized and actionable recommendations, generated by the rule-based engine based on the user-specific inputs represented in Fig 4. Conversely, a low risk result is accompanied by preventative advice based on the underlying risk factors that may be identified, for example, a high BMI as shown in Fig 5. This seamless and integrated workflow is a proof-of-concept of how high-accuracy diagnosis can be directly translated into concrete information for patient self-management, and act immediately.

### 3.3 Prototype demonstration and evaluation phase

The performance of the prediction model was assessed using a set of standard classification metrics widely adapted in healthcare applications. To define these metrics, the following terms are used:

a. True Positive (TP): The model correctly predicts a patient has diabetes.
b. True Negative (TN): The model correctly predicts a patient does not have diabetes.
c. False Positive (FP): The model incorrectly predicts a patient has diabetes (a “Type I error”).
d. False Negative (FN): The model incorrectly predicts a patient does not have diabetes (a “Type II error”).

The following metrics were used for evaluation:

- Accuracy: It is the proportion of all classifications that were correct, whether positive or negative. It is defined as:

$$\begin{array}{c} \text{Accuracy }=\;\frac{Number\;of\;correct\;classification\;instances}{Total\;number\;of\;instances} \end{array}$$

- Precision: It is a measure of the model’s reliability and is calculated as the ratio of true positives to all positive predictions.

$$\begin{array}{c} \text{Precision }=\;\frac{True\;Positive}{True\;Positive+False\;Positive} \end{array}$$

- Recall (Sensitivity): It measures how many of the actual positive cases were correctly identified by the model. It is important when missing a positive case (false negative) is more costly than false positives

$$\begin{array}{c} \text{Recall }=\;\frac{True\;Positive}{True\;positive+False\;Negative} \end{array}$$

- F1-Score: It is the harmonic mean of precision and recall. It is useful when we need a balance between precision and recall as it combines both into a single number. A high F1 score means the model performs well on both metrics. Its range is [0,1].

$$\begin{array}{c} \text{F1 }=\;\frac{\text{2}\;\times Precision\times Recall}{Precision+Recall} \end{array}$$

These metrics offer a thorough and effective assessment of the model’s performance level to ensure that the model can be utilized in a clinical setting. The demonstration and evaluation phase were intended to deliver empirical evidence needed to answer the study’s fundamental research questions. The phase consisted of a set of experiments focused on benchmarking model performance, as well as from examining the architecture of the system gives a novel, integrated approach for diabetes care.

#### 3.3.1 Answer to research questions.

The extensive empirical assessment undertaken in this research offers conclusive evidence to answer the research questions. To answer RQ1, the results show that the hybrid BiLSTM architecture offers significant improvement of diagnostic accuracy and utility in terms of robust 84.02% against the standalone benchmarks by effectively capturing complex non-linear feature dependency as further elaborated in Section 4. In order to answer RQ2, the work has validated the idea that predictive insights can be made available in an actionable way, through a rule-based expert engine; this logic is being implemented in the prototype (detailed in Section 3.2.3), which has been able to bridge the gap between passive diagnosis and proactive self-management.

## 4. Results and discussion

The performance of the proposed system was carefully assessed to provide robust performance, clinical reliability and high generalization ability. To this end, a leakage-safe 10-fold stratified cross-validation approach was used for all models. This methodology makes sure that data preprocessing such as imputation, scaling, and resampling are carried out strictly in each fold and therefore represent a realistic estimation of model’s performance using unseen clinical data. The experimental results presented in Table 1, it gives a detailed comparison of proposed biLSTM model against even the standard ML and DL architectures. The results show that the proposed system is found to reliably outperform and outclass traditional classifiers. Traditional ML models like Naive Bayes, KNN Models for the given dataset gave moderate performance results with the SVM and Decision Tree giving better results with 82.24% and 81.51% accuracy respectively. Within the DL suite the standard architectures such as RNN and LSTM were used which were competitive but superiority was found in BiLSTM model for diabetes prediction.

**Table 1:** Performance of all individual models.

Model	Accuracy (%)	Precision (%)	Recall (%)	F1-score (%)
**NB**	76.32	80.48	72.23	75.91
**CNN**	78.67	80.16	78.31	79.14
**KNN**	79.32	81.06	78.78	79.79
**DT**	81.51	82.30	82.36	82.23
**SVM**	82.24	85.86	78.93	82.16
**RNN**	82.56	85.19	80.65	82.76
**LSTM**	83.69	86.76	81.12	83.76
**Proposed (BiLSTM)**	84.02	87.89	80.50	83.91

**Fig 6:**
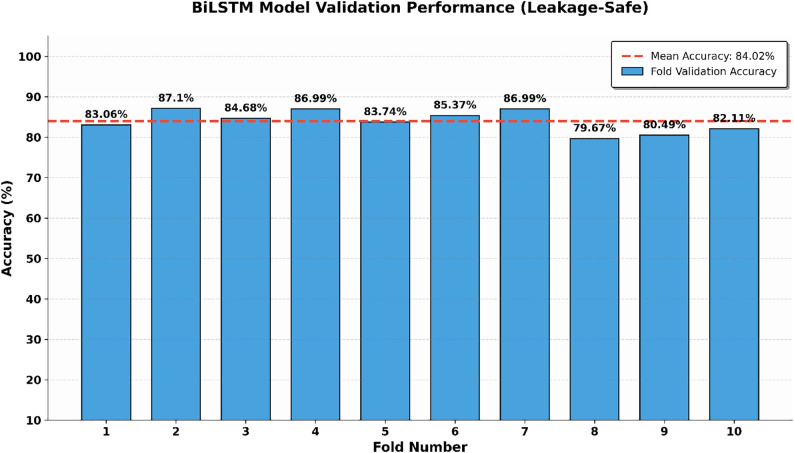
Cross-validation performance of the proposed BiLSTM Model.

**Fig 7:**
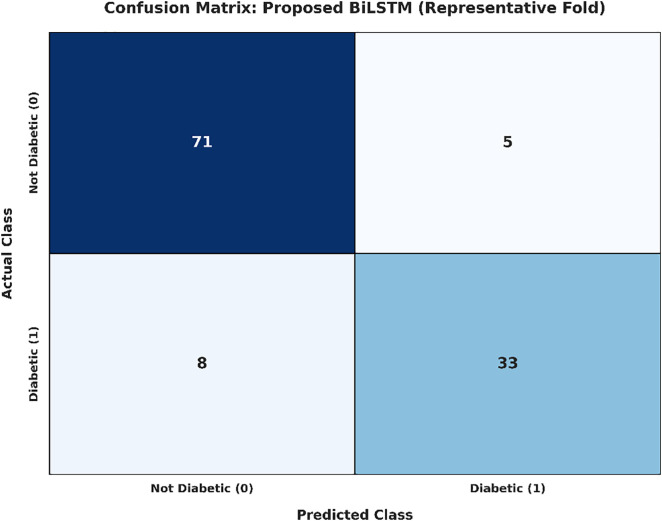
Confusion matrix of the BiLSTM model on a representative validation fold.

**Fig 8:**
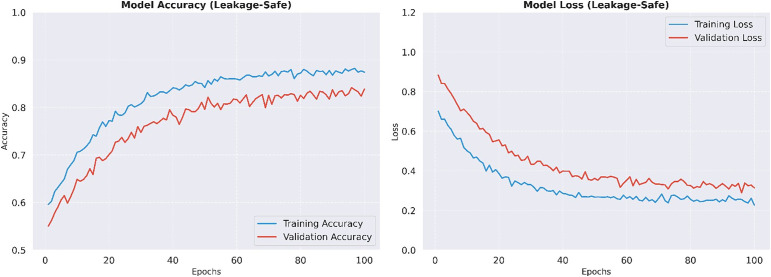
Graphical view of model training and validation performance (accuracy/loss curves).

The average cross-validated accuracy of the proposed BiLSTM model is 84.02% which is shown in Fig 6. While the margin for accuracy in comparison to the normal LSTM is in focus, the real punch of the BiLSTM architecture is it has high precision (87.89%). In a clinical setting, more accurate results are essential as “false alarms” are reduced and people with diabetes are not unnecessarily burdened with diabetic alerts. The bi-directional nature of the model enables it to reflect complex dependencies between clinical features from both forward and backward contexts and is an incredibly powerful feature extractor. A granular view of the classification of the model in a representative indication of a validation fold is described in the confusion matrix in Fig 7. The matrix shows the model’s specificity, which is high; the model also achieves a balance in the classification of diabetic and non-diabetic instances. Furthermore, the training and validation curves of Fig 8 confirm a stable convergence process. The small gap between the training and testing curves is empirical evidence of generalization ability of the model, meaning that the model learned meaningful clinical patterns instead of overfitting on the training subset.

### 4.1 Comparison with state of the art

As shown in Table 2, the comparative evaluation against literature however reveals a critical gap where the existing studies usually have prioritized theoretical metrics over clinical utility. For example, Hasan et al. [7] has a great risk of missed diagnosis due to lack of sensitivity, and Reza et al. [22] shows poor generalization performance using real clinical data. The proposed hybrid framework provides a better diagnostic profile with a high accuracy of 84.02% and high precision of 87.89% using a strict partitioned validation protocol. By using the BiLSTM architecture, the model is able to decipher complex and non-linear physiological dependencies missed by the traditional ML models, thus reducing false alarms and allowing for more confidence by the practitioner. Furthermore, this research is different as it eliminates the diagnostic dead end that was prevalent in studies such as Dejneka et al. [23] which was limited to passive classification. By combining a high performance DL engine and an automated rule based expert system the framework is able to move beyond the simple prediction to deliver personalized, actionable intervention [24,25].

**Table 2:** Comparative analysis of existing diabetes prediction and recommendation systems.

References	Methodology	Dataset	Performance metrics	Key contributions / Limitations
**Dutta et al.** [10]	ML Ensemble	South Asian Dataset	Acc: 73.5%, AUC: 0.83	Constrained to specific regional demographics; yields moderate accuracy without prescriptive clinical utility.
**Reza et al.** [22]	Stacking (Classical + DL)	PIMA Indians	Acc: 77.10%, AUC: 0.84	High performance (95%) is contingent upon simulated data; predictive efficacy significantly diminishes on authentic clinical cohorts.
**Dejene et al.** [23]	Classical ML (RF, XGB)	CDC Dataset	Acc: 83.3%	Operates on a macroscopic scale but functions strictly as a passive classifier, offering no actionable patient interventions.
**Sajid et al.** [16]	ML Ensemble + DL (RBM)	Fused PIMA & Food	F1-Score: 84.1%	Integrates dietary mapping, but the diagnostic core relies on traditional ML, potentially missing complex non-linear physiological interactions.
**Hasan et al.** [7]	Weighted Soft Voting	PIMA Indians	Recall: 78.9%, AUC: 0.95	Prioritizes AUC optimization, leading to a pronounced sensitivity deficit (low recall) which elevates the risk of false negatives in screening.
**Proposed Model**	**Integrated BiLSTM & Rule-Based System**	**Fused (PIMA + Type 2)**	**Acc: 84.02%, Prec: 87.89%, Rec: 80.50%, F1: 83.91%**	**Validates via a strictly partitioned protocol; leverages deep feature extraction; provides an automated, personalized health guidance module.**

## 5. Conclusion and future work

This study has successfully solved the issue of chronic disease management by proposing an integrated system designed specifically for diabetes management. This was done through overcoming the key constraint of data scarcity by generating a new, fused dataset, which was subsequently operated in a robust, leakage safe pipeline to balance and generalize it. The most significant contribution is the development of the BiLSTM model that was evaluated against the classic ML-based classifiers (Decision Tree, SVM, KNN, Naive Bayes), as well as standard DL models (RNN, CNN, LSTM). The outcomes of this evaluation confirm the higher predictive potential of the BiLSTM with an accuracy of 84.02%. By integrating this predictive engine with a rule based recommender system and embedding this into a working prototype, this study offers a holistic tool that presents a valuable bridge between a calculated clinical risk score and a self management plan adopted by a patient. Future work will include incorporation of real-time wearable data, reinforcement learning to receive more valuable recommendations, and making the system transparent through XAI.
